# Case of Secondary Tics Associated With Olanzapine in an Adult

**DOI:** 10.3389/fpsyt.2017.00150

**Published:** 2017-08-15

**Authors:** Eric W. Mills, Lindsay S. Shaffer, Fernando S. Goes, Akira Sawa, Frederick C. Nucifora

**Affiliations:** ^1^Department of Psychiatry and Behavioral Sciences, Johns Hopkins University School of Medicine, Baltimore, MD, United States

**Keywords:** olanzapine, atypical antipsychotic, neuroleptic, tics, schizophrenia

## Abstract

Atypical antipsychotic medications, such as risperidone, aripiprazole, and olanzapine, have utility in treating motor tics, particularly in Tourette syndrome. In rare cases, atypical antipsychotic medications have been associated with adult-onset motor tics. Such adverse drug reactions have been documented in response to quetiapine, aripiprazole, and amisulpride. Here, we report, to our knowledge, the first case of adult-onset motor tics related to olanzapine administration.

## Introduction

A 22-year-old man diagnosed with schizoaffective disorder, manic type, with a history of autism was admitted to our inpatient unit after presenting to the emergency room with mood and psychotic symptoms in the setting of medication non-compliance. His psychiatric history is notable for periods of increased mood, lack of sleep, grandiosity, and religious delusions. He also had episodes of depression and a history of a suicide attempt. However, his perceptual disturbances, including auditory hallucinations and delusions, were chronic and occurred even at times when his mood was euthymic. Therefore, he was formulated has having schizoaffective disorder. He presented to the emergency room with auditory hallucinations, delusional thinking, disorganized behavior, and elevated mood. Prior to this presentation, these symptoms were mostly controlled by risperidone 5 mg daily and lithium 300 mg daily. However, he stopped taking his medications prior to this episode and decompensated. On hospital admission, risperidone and lithium were restarted.

While under our inpatient care, the patient developed asymptomatic hyperprolactinemia, which was suspected to be due to risperidone. For this reason, and that he still had some persistence of psychotic symptoms, risperidone was tapered over the course of 5 days and olanzapine was initiated and titrated up to 10 mg twice daily in addition to 600 mg lithium twice daily. He endorsed better control of his symptoms on olanzapine than risperidone, and his mood was euthymic, but he still had some minor hallucinations and delusional thinking.

Ten days after the first dose of olanzapine (and 5 days after the last dose of risperidone), the patient developed frequent, sudden, non-rhythmic movements of his left face, neck, and shoulder consistent with the appearance of a simple motor tic. These movements did not have any of the distinctive appearances of the rhythmic or writhing nature of choreiform movements nor features of dystonic movements or fixed posturing. He also did not exhibit any myoclonic movements or tardive dyskinesia. His movements were suppressible, partially distractible, and clearly associated with premonitory sensation(s). We used the Yale global tic severity scale (YGTSS) to quantify the severity of tic-like movements in our patient (1), and his symptoms were given a score of 38 (of a total of 50). Neither our patient nor his caregivers described any prior appearance of abnormal movements or tic-like behaviors. On consultation of the medical record, there was no family history of tics noted.

Given that the patient’s new symptoms coincided with the administration of olanzapine, we considered a possible adverse drug reaction to olanzapine and decided to cross-taper medications. Due to the fact that the patient had side effects or only a partial response to several other antipsychotics on this admission and in the past, the superior efficacy of clozapine in treating psychosis, and the fact that clozapine is less likely to cause movement disorders than other antipsychotics, olanzapine was cross tapered to clozapine. Over 10 days, olanzapine was tapered and clozapine was initiated at 50 mg daily, increasing to 250 mg daily over 14 days. On the 20th day, the YGTSS score was 21/50. Seven days after the last dose of olanzapine (day 27), the YGTSS score was 0/50. During this cross-taper, the patient’s lithium regimen was continued unchanged. Given that the patient’s symptoms abated following discontinuation of olanzapine (while still receiving lithium), it is unlikely that these new symptoms reflect a lithium-induced movement disorder. On this new regimen of clozapine and lithium, his psychotic symptoms were adequately controlled, and no abnormal movements were present. The patient was discharged with close follow-up. No recrudescence of the described movement symptoms has occurred since that time.

## Background

Interestingly, antipsychotic medications, particularly the first-generation or typical antipsychotic medications, have been used to treat tics in Tourette’s syndrome (2–5). Unfortunately, these medications can cause movement disorders such as akathisia, dystonia, extrapyramidal symptoms, and tardive dyskinesia. The development of the second-generation or atypical antipsychotics, which tend to have less movement disorders associated with their use, hold promise in also treating tics. The first report of olanzapine reducing tics was in a 16-year-old female who had onset of Tourette’s syndrome at age 5 (6). She had failed haloperidol, pimozide, and risperidone due to ineffectiveness or side effects. By 9 weeks of treatment with olanzapine, her tics were partially controlled. Subsequently, several open-label studies in children and adults have assessed the effect of olanzapine on tic severity in Tourette’s syndrome (7–11). While these studies were limited in the number of patients enrolled, typically between 10 to 14 patients, all studies showed a significant improvement in tics by the YGTSS. In addition, while most of these studies were for a short duration of 6–10 weeks, Budman et al. assessed patients after 6 months and three patients continued to have improvement. In another study, olanzapine was compared to pimozide in a 52-week double-blind cross-over study and showed that olanzapine was superior to pimozide in reducing tics (12). Taken together, these studies suggest that olanzapine could be used as a treatment for tics. In contrast, drug-induced motor tics is a rare condition, and its underlying mechanism is poorly understood. At least three cases of tics associated with atypical antipsychotic medications, including quetiapine, aripiprazole, and amisulpride, have been reported previously, but do not include olanzapine (13–15). Two of these cases involved treatment for schizophrenia and the third for bipolar disorder. Large studies of adverse drug effects, including those for olanzapine, have not identified tics as a common side effect of neuroleptics (16). However, since olanzapine could be used to treat tics, it is important to recognize that olanzapine even if in rare cases could potentially cause tics. Furthermore, the wide use of olanzapine and atypical antipsychotics in general necessitates the awareness of these potential side effects for this class of medications.

## Discussion

In this case report, we describe an adult patient with a new onset of a movement disorder resembling motor tics. Initial diagnostic consideration for adult-onset of tic-like movements includes a number of distinct conditions that must be excluded. Adult-onset tics may be most commonly caused by a general medical condition such as stroke or ingestion of psychoactive compounds such as illicit drugs. Such considerations, undoubtedly, are important in forming diagnostic impressions early on. Our patient lacked any concurrent medical illness, which might explain his new symptoms, no history of substance use, and drug screening on admission was negative for any illicit substances.

“Unmasked” primary tics or Tourette’s syndrome should also be considered particularly since the patient’s movement symptoms developed shortly after discontinuing risperidone. Consultation with family members and prior care providers is crucial to determine whether there may be an occult history of tics or tic-like behaviors. Our patient has no personal or family history of movement disorders or tic disorders. Moreover, our patient did not develop tics during previous medication-free intervals, supporting the impression that he does not have a primary tic disorder. An important caveat, however, is that primary tics may display a variable, fluctuating course. As such, a primary tic disorder cannot be definitively excluded. The tics occurring from withdrawal from risperidone is also a possibility. However, he had not been compliant with risperidone prior to admission and had no evidence of tics upon his initial presentation.

Finally, secondary causes of tics must be distinguished from a psychogenic movement disorder resembling tics (PMRTs) (17). PMRTs (or “functional” tics) are more commonly suggestible and less likely to be associated with premonitory sensations (18). Our patient’s symptoms were not readily suggestible and clearly were associated with premonitory discomfort, suggesting a diagnosis of secondary tics rather than PMRT. That the patient’s symptoms abated following discontinuation of olanzapine and initiation of clozapine also provides supportive evidence that the patient’s symptoms were not psychogenic in nature. However, PMRT cannot be entirely excluded.

## Concluding Remarks

Taken together, this report supports the emerging diversity of movement disorders that may complicate neuroleptic use in psychiatric patients.

## Ethics Statement

This study was carried out in accordance with the recommendations of Institutional Review Board of Johns Hopkins University School of Medicine with written informed consent from all subjects. All subjects gave written informed consent in accordance with the Declaration of Helsinki.

## Author Contributions

EM, FG, and FN contributed to the clinical care. EM, LS, FG, AS, and FN contributed to the discussion of the case and wrote the case report.

## Conflict of Interest Statement

The authors declare that the research was conducted in the absence of any commercial or financial relationships that could be construed as a potential conflict of interest.
